# Life Histories of the Seed Bugs, *Kleidocerys punctatus* and *Kleidocerys virescens*

**DOI:** 10.1673/031.010.9101

**Published:** 2010-07-05

**Authors:** Luis Cervantes Peredo, Marcela Briceño Baez

**Affiliations:** Instituto de Ecologia, A.C. Km 2.5 Antigua Carretera a Coatepec # 351, Xalapa, Veracruz, Mexico

**Keywords:** *Alnus*, *Buddleia*, *Nicotiana*, Mexico, Heteroptera: Lygaeidae

## Abstract

The life cycles of the seed bugs, *Kleidocerys punctatus* Distant and *Kleidocerys virescens* F. (Hemiptera: Lygaeidae: Ischnorhynchinae), are reported for the first time. Description of all immature stages and adults are included. Adults and nymphs of *K. punctatus* are associated with several species of *Alnus* (Betulaceae), while those of *K. virescens* are associated with *Nicotiana glauca* Graham, *Nicotiana tabacum* L. (Solanaceae), and *Buddleia crotonoides* A. Gray and *Buddleia* sp. (Loganiaceae). Adults and nymphs feed mainly on the seeds, inside the dry fruit, but they also take plant juices from other reproductive and vegetative structures. Illustrations of the eggs, all nymphal instars, and the adults, as well as notes on their biology and their distribution in Mexico, are included.

## Introduction

Species in the genus *Kleidocerys* (Hemiptera: Lygaeidae: Ischnorhynchinae) are widely distributed in the Palearctic, Nearctic and Neotropical Regions. On the American Continent, eleven species have been reported: *K. denticollis, K. dimidiatus, K. franciscanus, K. modestus, K. obovatus, K. ovalis, K. pallipes, K. punctatus* Distant, *K. resedae, K. suffussus, K. virescens* F., with two subspecies *K. resedae fuscomaculatus* and *K. resedae geminatus* (Barber 1953; Scudder 1962, Brailovsky 1976). For Mexico, only four species have been registered *K. pallipes, K. punctatus, K. resedae geminatus*, and *K. virescens* (Brailovsky 1976). *Kleidocerys resedae* is one of the best known species; Claassen (1921) and Wheeler (1976) have studied its life history, and the latter reported 43 species of host plants. Leston (1957) mentioned a stridulatory mechanism for this species. Southwood and Leston (1959) described the life cycle of *K. ericae*. Scudder (1962) summarized the known biology and host plant records for *Kleidocerys* species. *Kleidocerys virescens* has been recorded as associated with *Scoparia dulcis* in Florida, and in Martinique, it was collected on *Lobelia viridiflora* (Slater and Baranowski 1990).

Here, the life histories of *K. punctatus* and *K. virescens* are described, illustrations of eggs, all nymphal instars (except first and second instars of *K. punctatus*), and adults are provided, and notes are given on their biology and distributions in Mexico.

## Materials and Methods

Alive material was collected in the Mexican states of Guerrero, Oaxaca, and Puebla from 2004 to 2008. Other specimens were obtained from the Entomological Collection of Instituto de Ecologia, A.C. (IEXA) and from the National Collection of Insects of Instituto de Biologia, UNAM (CNIN).

Bugs were collected directly from the host plants with an aspirator. Insects were kept alive in 10 × 8 × 8 cm plastic containers under laboratory conditions; a humid cotton ball and fresh fruits of its host plant were added every three days, and eggs were usually removed from the plant tissue or the walls of the container and put in small pieces of humid cotton. Drawings were made using a drawing tube adaptor, and measurements are given in millimeters.

## Results

**Plate 1.**
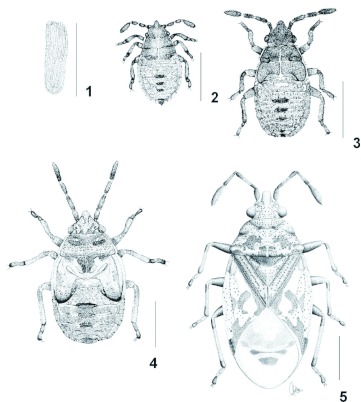
Figures 1–5. Life stages of *Kleidocerys punctatus*. 1. Egg. 2. Third instar. 3. Fourth instar. 4. Fifth instar. 5. Adult. High quality figures are available online.

***Kleidocerys punctatus* (Distant) (Plate 1. Figures 1–5.)**
**Egg** (Figure 1). Elongated, with posterior pole slightly pointed, anterior pole rounded and surrounded by a crown of micropylar process. Corium with longitudinal and slightly elevated crests. Yellow when laid, and they turned reddish in three to four days. Length 0.98 ± 0.03; width 0.31 ± 0.03 (*n* = 10).
**Third Instar** (Figure 2). Pyriform, with abdomen much wider than pronotum, some individuals with abdomen more than twice the width across the anterior margin of pronotum. Head, pro-, meso-, metanotum, antennae, rostrum, legs, and abdominal scent gland plates of segments III–IV, IV–V, V–VI, dorsal mesial plates of segments VIII and IX, and ventral mesial plates of sternites VI to IX brown. Antennae and molting sutures slightly reddish. Abdomen variegated with red and pale yellow. Rostrum reaching metacoxae. Spiracles apparent and situated more posterior on each segment of the lateral margin.
  Measurements (*n* = 10). Body length 1.56 ± 0.14; head length 0.36 ± 0.04; width across eyes 0.46 ± 0.02; interocular distance 0.33 ± 0.04; antennal segments: I 0.1 ± 0.02, II 0.15 ±0 .01, III 0.13 ± 0.01, IV 0.2 2 ± 0.01; rostral segments: I 0.22 ± 0.03, II 0.2 ± 0.02, III 0.21 ± 0.01, IV 0.19 ± 0.03; pronotum: length 0.36 ± 0.07, width across humeral angles 0.7 ± 0.03; hind leg: femur length 0.33 ± 0.04, tibia length 0.39 ± 0.02, length of tarsal segments: I 0.07 ± 0.01, II 0.13 ± 0.03.
**Fourth Instar** (Figure 3). Pyriform. Very similar to third instar, although the setigerous punctures on anterior part of head, pro-, meso, metanotum were more apparent. Lateral margins of pro-, and mesonotum expanded and pale yellow. Rostrum reaching metacoxae. Mesothoraxic wing pads reaching first abdominal segment.
  Measurements (*n* = 10). Body length 2.15 ± 0.13; head length 0.41 ± 0.08; width across eyes 0.58 ± 0.02; interocular distance 0.4 ± 0.01; antennal segments: I 0.13 ± 0.02, II 0.23 ± 0.03, III 0.19 ± 0.02, IV 0.32 ± 0.03; rostral segments: I 0.28 ± 0.03, II 0.29 ± 0.04, III 0.28 ± 0.03, IV 0.25 ± 0.02; pronotum: length 0.26 ± 0.02, width across humeral angles 0.81±0.03; hind leg: femur length 0.42 ± 0.03, tibia length 0.49 ± 0.04, length of tarsal segments: I 0.08 ± 0.03, II 0.19 ± 0.03.
**Fifth Instar** (Figure 4). Pyriform, with maximum width across abdominal segment IV. Head, pro-, meso-, metanotum variegated with brown, red, and pale yellow; usually bases of head, pronotum, mesonotum, and apex of wing pads darker, mainly brown. Posterior part of pronotum, disc of wing pads, and apex of scutellum lighter, usually pale yellow or red. A well defined dark brown line along the submarginal border of pro-, and mesonotum. Antennal segments brown, segments I to III each with base and apex reddish; joint of segments I–II reddish and those of segments II–III, and III–IV whitish; central areas of segments II and III sometimes reddish. Abdomen variegated with red and yellow or creamy white; usually the paler areas were along segmental joints and in the punctures. Rostrum reaching abdominal sternite I. Mesothoraxic wing pads reaching the middle part of abdominal segment II.
  Measurements (*n* = 10). Body length 3.06 ± 0.3; head length 0.43 ± 0.09; width across eyes 0.74 ± 0.03; interocular distance 0.44 ± 0.03; antennal segments: I 0.16 ± 0.02, II 0.34 ± 0.03, III 0.28 ± 0.02, IV 0.45 ± 0.03; rostral segments: I 0.38 ± 0.05, II 0.43 ± 0.05, III 0.41 ± 0.03, IV 0.27 ± 0.01; pronotum: length 0.4 ± 0.05, width across humeral angles 1.18 ± 0.06; hind leg: femur length 0.66 ± 0.1, tibia length 0.65 ± 0.1, length of tarsal segments: 10.13 ± 0.02, II 0.2 ± 0.04.
**Adult** (Figure 5). Head pale brown, pronotum brown, hemelytra yellowish brown, all coarsely punctured. Most of antennal segments ochraceous, basal segment brown with apex black, bases and apices of segment II and III and terminal segment (except base) dark brown. Rostrum extended to first sternite. Scutellum about one fourth wider than long. Corium, excluding lateral margins clouded with dark brown, with a transverse discal spot and the apex blackish; the clavus and sublateral areas very coarsely punctuate. Membrane grayish, with basal area black. Body beneath with the abdomen ochraceous, coarsely punctured. Legs brownish red, with apex of femora slightly paler.
  Female Measurements (*n* = 10). Body length 4.1 ± 0.13; head length 0.41 ± 0.06; width across eyes 0.8 ± 0.02; interocular distance 0.42 ± 0.03; antennal segments: I 0.24 ± 0.05, II 0.58 ± 0.04, III 0.42 ± 0.03, IV 0.5 ± 0.04; rostral segments: I 0.48 ± 0.02, II 0.57 ± 0.05, III 0.54 ± 0.09, IV 0.29 ± 0.04; pronotum: length 0.84 ± 0.04, width across humeral angles 1.57 ± 0.04; scutellum: length 0.62 ± 0.04, width 0.88 ± 0.04; hind leg: femur length 1.04 ± 0.07, tibia length 1.05 ± 0.1, length of tarsal segments: I 0.18 ± 0.02, II 0.09 ± 0.01, III 0.15 ± 0.02.
  Male Measurements (*n* = 10). Body length 3.89 ± 0.17; head length 0.42 ± 0.08; width across eyes 0.76 ± 0.03; interocular distance 0.4 ± 0.03; antennal segments: I 0.24 ± 0.03, II 0.56 ± 0.04, III 0.44 ± 0.04, IV 0.49 ± 0.04; rostral segments: I 0.5 ± 0.04, II 0.52 ± 0.05, III 0.52 ± 0.03, IV 0.3 ± 0.02; pronotum: length 0.78 ± 0.06, width across humeral angles 1.48 ± 0.03; scutellum: length 0.61 ± 0.08, width 0.81 ± 0.04; hind leg: femur length 0.97 ± 0.07, tibia length 0.98 ± 0.09, length of tarsal segments: I 0.18 ± 0.02, II 0.09 ± 0.01, III 0.17 ± 0.02.
**Biology.** *Kleidocerys punctatus* has been collected on several species of *Alnus* (Betulaceae). Adults and nymphs feed on the seeds of mature fruits. In one occasion, it was found associated with *Penstemon barbatus* (Cav.) Roth (Scrophulariaceae).
  Eggs are normally deposited between the scales of the cones of *Alnus* spp., and nymphs and adults are usually strongly attached to cones and stems. They are typically present just when the trees are producing cones (April–May), so it is probable that they are univoltine.
**Distribution.** Mexico: Hidalgo, Michoacan, Oaxaca, Puebla, Queretaro, State of Mexico, Veracruz.

**Plate 2.**
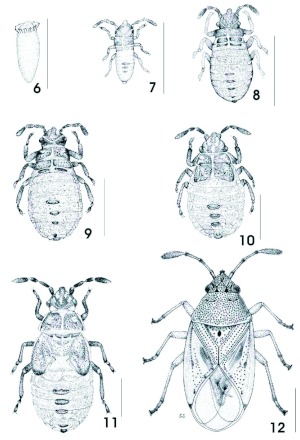
Figures 6–12. Life stages of *Kleidocerys virescens*. 6. Egg. 7. First Instar. 8. Second Instar. 9. Third instar. 10. Fourth instar. 11. Fifth instar. 12. Adult. High quality figures are available online.

***Kleidocerys virescens* (Fabricius) (Plate 1. Figures 6–12)**
**Egg** (Figure 6). Elongated with posterior pole slightly pointed, anterior pole rounded, surrounded by micropylar processes in form of crown. Yellow when laid, turning red as the embryo develops. Length 0.92 ± 0.06; width 0.28 ± 0.03 (*n* = 10).
**First Instar** (Figure 7). Elongated, body cylindrical, with lateral margins nearly parallel and slightly serrated due to the divisions of abdominal segments. Head, antennae, rostrum, thorax, legs, and scent gland plates of abdominal segments III–IV, IV–V, and V–VI pale brown. Eyes and abdomen reddish. Rostrum reaching abdominal sternite III.
  Measurements (*n* = 2). Body length 0.91 ± 0.04; head length 0.28 ± 0.03; width across eyes 0.26 ± 0; interocular distance 0.2 ± 0; antennal segments: I 0.07 ± 0.01, II 0.07 ± 0.01, III 0.09 ± 0.01, IV 0.14 ± 0.08; rostral segments: I 0.14 ± 0.06, II 0.16 ± 0.06, III 0.16 ± 0.06, IV 0.16 ± 0.03 ; pronotum: length 0.1 ± 0, width across humeral angles 0.26 ± 0; hind leg: femur length 0.2 ± 0, tibia length 0.19 ± 0.04, length of tarsal segments: I 0.04 ± 0.03, II 0.12 ± 0.03.
**Second Instar** (Figure 8). Slightly pyriform; very similar to the first instar although the abdomen was much wider and was variegated with red and pale yellow. Scent gland openings similar as in first instar, although more apparent. Rostrum reaching abdominal sternite II.
  Measurements (*n* = 10). Body length 1.22 ± 0.16; head length 0.31 ± 0.03; width across eyes 0.35 ± 0.01; interocular distance 0.26 ± 0.01; antennal segments: I 0.08 ± 0.01, II 0.12 ± 0.01, III 0.1 ± 0.01, IV 0.22 ± 0.01; rostral segments: I 0.17 ± 0.03, II 0.18 ± 0.04, III 0.16 ± 0.01, IV 0.19 ± 0.02; pronotum: length 0.12 ± 0.02, width across humeral angles 0.45 ± 0.06; hind leg: femur length 0.23 ± 0.08, tibia length 0.22 ± 0.04, length of tarsal segments: I 0.05 ± 0.01, II 0.11 ± 0.02.
**Third Instar** (Figure 9). Very similar to second instar, although the punctures on thorax were more visible. Rostrum reaching abdominal sternite I.
  Measurements (*n* = 10). Body length 1.76 ± 0.17; head length 0.33 ± 0.05; width across eyes 0.48 ± 0.06; interocular distance 0.34 ± 0.04; antennal segments: I 0.11 ± 0.02, II 0.18 ± 0.02, III 0.15 ± 0.03, IV 0.26 ± 0.03; rostral segments: I 0.24 ± 0.03, II 0.28 ± 0.05, III 0.25 ± 0.04, IV 0.23 ± 0.03; pronotum: length 0.18 ± 0.05, width across humeral angles 0.64 ± 0.09; hind leg: femur length 0.33 ± 0.08, tibia length 0.3 ± 0.06, length of tarsal segments: 10.08 ± 0.01, II 0.13 ± 0.02.
**Fourth Instar** (Figure 10). Pyriform, with maximum width across abdominal segment III. Head pale brown, with lateral margins and margin of tylus slightly darker, molting suture reddish; antennae and legs brown with some reddish areas; pro-, meso-, and metanotum variegated with brown, red and pale yellow; lateral margins of pro-, and mesonotum pale yellow and slightly expanded; pro-, meso-, and metapleura pale brown; abdomen variegated with red and pale yellow, with the pale yellow areas usually as row of punctures. Abdominal plates on segments III–IV, IV–V, V–VI, VIII, and IX, the first three with the scent gland openings. Sternites VII–IX with small, pale brown plates situated mesially. Rostrum reaching sternite I. Meso and metathoraxic wind pads covering part of abdominal segment I. Area in front of eyes, pro-, meso, metanotum and abdomen covered by setigerous punctures with very small setae.
  Measurements (*n* = 10). Body length 2.21 ± 0.07; head length 0.42 ± 0.08; width across eyes 0.59 ± 0.04; interocular distance 0.42 ± 0.02; antennal segments: I 0.15 ± 0, II 0.22 ± 0.02, III 0.18 ± 0.02, IV 0.31 ± 0.02; rostral segments: I 0.29 ± 0.02, II 0.34 ± 0.04, III 0.3 ± 0.01, IV 0.28 ± 0.02; pronotum: length 0.26 ± 0.04, width across humeral angles 0.77 ± 0.05; hind leg: femur length 0.46 ± 0.06, tibia length 0.49 ± 0.08, length of tarsal segments: I 0.1 ± 0.01, II 0.15 ± 0.02.
**Fifth Instar** (Figure 11). Slightly pyriform, with maximum width across abdominal segment III. Head, pronotum, mesothoraxic wing pads, and scutellum variegated with brown, red, and pale yellow; apex of head slightly darker; lateral margins of pronotum and wing pads slightly expanded and pale yellow, a brown line just after the expanded lateral margins. Antenna, rostrum, and legs dark brown; joints between antennal segments pale yellow; and last antennal segment slightly reddish. Abdomen variegated with red and pale yellow; openings of scent glands situated on segments III–IV, IV–V, and V–VI as pale brown plates; other pale brown plates mesially on segments VIII and IX. Abdominal sternites VI–IX with small rectangular, brown, medial plates. Area in front of eyes, pronotum, wing pads, scutellum, and abdomen covered by setigerous punctures, setae small, silvery colored, and directed backwards. Rostrum reaching abdominal sternite I.
  Measurements (*n* = 10). Body length 3.16 ± 0.25; head length 0.49 ± 0.05; width across eyes 0.76 ± 0.05; interocular distance 0.52 ± 0.03; antennal segments: I 0.2 ± 0.04, II 0.3 ± 0.03, III 0.22 ± 0.02, IV 0.45 ± 0.02; rostral segments: I 0.43 ± 0.04, II 0.42 ± 0.04, III 0.43 ± 0.03, IV 0.32 ± 0.04; pronotum: length 0.43 ± 0.04, width across humeral angles 1.14 ± 0.05; hind leg: femur length 0.68 ± 0.08, tibia length 0.66 ± 0.08, length of tarsal segments: 10.14 ± 0.03, II 0.2 ± 0.03.
**Adult** (Figure 12). Head and pronotum pale ochraceous, thickly covered with dark punctures. Hemelytra yellowish brown. Most of antennal segments ochraceous, basal segment brown, bases of segments III and IV dark brown. Rostrum extended to posterior coxae. Scutellum pale ochraceous, thickly, coarsely, and darkly punctuated at base, a few coarse dark punctures along lateral margins, and a central elongated dark spot. Corium, pale ochraceous, with two dark spots on disk and four along apical margin. Femora and apices of tibiae castaneous, tibiae and apices of femora pale ochraceous, tarsi ochraceous.
  Female Measurements (*n* = 10). Body length 3.24 ± 0.17; head length 0.42 ± 0.06; width across eyes 0.7 ± 0.03; interocular distance 0.42 ± 0.03; antennal segments: I 0.2 ± 0, II 0.4 ± 0.03, III 0.3 ± 0.03, IV 0.5 ± 0.05; rostral segments: I 0.38 ± 0.06, II 0.43 ± 0.06, III 0.46 ± 0.05, IV 0.28 ± 0.05; pronotum: length 0.72 ± 0.04, width across humeral angles 1.23 ± 0.09; scutellum: length 0.42 ± 0.03, width 0.62 ± 0.05; hind leg: femur length 0.8 ± 0.03, tibia length 0.82 ± 0.04, length of tarsal segments: I 0.11 ± 0.02, II 0.07 ± 0.01, III 0.14 ± 0.02.
  Male Measurements (*n* = 10). Body length 3.2 ± 0.18; head length 0.4 ± 0.05; width across eyes 0.69 ± 0.04; interocular distance 0.4 ± 0.03; antennal segments: I 0.2 ± 0, II 0.42 ± 0.03, III 0.32 ± 0.03, IV 0.48 ± 0.03; rostral segments: I 0.38 ± 0.06, II 0.4 ± 0.06, III 0.48 ± 0.05, IV 0.28 ± 0.04; pronotum: length 0.69 ± 0.05, width across humeral angles 1.16 ± 0.07; scutellum: length 0.38 ± 0.04, width 0.6 ± 0.05; hind leg: femur length 0.78 ± 0.05, tibia length 0.84 ± 0.08, length of tarsal segments: I 0.13 ± 0.02, II 0.08 ± 0.01, III 0.13 ± 0.02.
**Biology.** *Kleidocerys virescens* was found on several occasions feeding on *Nicotiana glauca* Graham, *Nicotiana tabacum* L. (Solanaceae), *Buddleia crotonoides* A. Gray and *Buddleia* sp. (Loganiaceae). *Nicotiana glauca* is an invasive species that is usually found along roads, especially where the soil has been disturbed. Consequently this plant and this associated bug are widely distributed in Mexico. Specimens were found inside the dry inflorescences, feeding on the seeds; adults were sometimes found on fresh flowers and fruits where they were feeding on the juices from these structures. They are present year around, so they probably have more than one generation each year.
**Distribution.** Mexico: Campeche, Chiapas, Colima, Distrito Federal, Guanajuato, Guerrero, Jalisco, Michoacan, Morelos, Nayarit, Nevo Leon, Oaxaca, Puebla, Quintana Roo, San Luis Potosi, Sinaloa, Tabasco, Veracruz, Yucatan.

## Discussion

Most other known biological information on members of *Kleidocerys* comes mainly from the species *Kleidocerys resedae* (Claassen 1921; Wheeler 1976), *K. ericae* (Southwood and Leston 1959), and by isolated reports of host plants of other species (Scudder 1962). Here, the nymphs of *K. punctatus* (an arboreal species mainly associated with *Alnus* spp. that may also use herbaceous plants in the genus *Penstemon* as an alternative host) and of *K. virescens* (a species associated with shrubs in the families Solanaceae and Loganiaceae) are described. Some of these records are related to the ones reported in the literature (see Scudder 1962), although reports in the Solanaceae and Loganiaceae are new. A revision of the genus *Kleidocerys* for Mexico is in progress and these records will confirm the reports of hosts of other known and new species.

All the nymphs of the species known in the genus *Kleidocerys* have three paired dorsal abdominal scent gland orificies. Nymphs of different species can be differentiated mainly by coloration, size, and the distribution of punctures and the transverse dark bands of the pronotum. *Kleidocerys punctatus* has the pronotal bands darker than *K. virescens*; *K. virescens* has the apex of the head slightly darker and has more punctures on the hemelytra than *K. punctatus. Kleidocerys punctatus* has a wider body than *K. virescens*.

The distribution of *K. punctatus* is restrained to the areas of cloud forest and oak-pine forest where its host plant is present; while the distribution of *K. virescens* is very wide, due to the extensive distribution of its host plants, specially *Nicotiana glauca*.
